# Tumor Mutational Burden–High Intrahepatic Cholangiocarcinoma Presenting with Solitary Brain Metastasis: A Case of Precision Oncology

**DOI:** 10.70352/scrj.cr.25-0620

**Published:** 2026-01-08

**Authors:** Takahiro Maehata, Yuta Ushida, Gen Sugawara, Yoriko Yamashita, Masaya Inoue

**Affiliations:** 1 Department of Surgery, Toyota Kosei Hospital, Toyota, Aichi, Japan; 2 Department of Pathology, Toyota Kosei Hospital, Toyota, Aichi, Japan

**Keywords:** cholangiocarcinoma, biliary tract cancer, solitary brain metastasis, tumor mutational burden–high

## Abstract

**INTRODUCTION:**

Brain metastasis from intrahepatic cholangiocarcinoma (ICC) is a rare condition with a poor prognosis, and no standard treatment has been established. This report aims to present a case of solitary ICC brain metastasis successfully treated with a multimodal approach guided by comprehensive genomic profiling (CGP).

**CASE PRESENTATION:**

A 64-year-old man, who had undergone a left hepatectomy for ICC 15 months prior, presented with recent memory difficulties. A brain MRI revealed a solitary 39-mm ring-enhancing mass in the left temporal lobe. The patient underwent surgical resection of the brain tumor, and histological examination confirmed the lesion was a metastasis from the primary ICC. Postoperatively, he received systemic therapy consisting of gemcitabine, cisplatin, and durvalumab. CGP on the resected brain specimen revealed a high tumor mutational burden status (23 mutations/Mb) and microsatellite stability. At the 8-month follow-up after the craniotomy, the patient remains disease-free with no signs of recurrence.

**CONCLUSIONS:**

This case suggests that an integrated approach, combining aggressive local therapy with systemic immunotherapy informed by biomarkers, can achieve a favorable outcome in selected patients with ICC. The identification of a high tumor mutational burden was crucial in guiding treatment and supports its potential as a predictive biomarker. This precision oncology strategy may improve the poor prognosis associated with this condition.

## Abbreviations

BTC
: biliary tract cancer
CA19-9
: carbohydrate antigen 19-9
CGP
: comprehensive genomic profiling
ICC
: intrahepatic cholangiocarcinoma
ICI
: immune checkpoint inhibitor
PD-L1
: programmed death–ligand 1
TMB
: tumor mutational burden
UICC
: Union for International Cancer Control

## INTRODUCTION

BTCs, including ICC, are aggressive malignancies with a poor prognosis, particularly in metastatic settings. Management of these cancers is challenging, partly because of their underlying molecular complexity.^1,2)^ ICC metastases commonly occur in the liver, regional lymph nodes, and lungs; the brain is considered a rare site of involvement.^3)^ Given the rarity of brain metastases from ICC, no standard care has been established. Therefore, a multimodal approach combining effective local therapy, such as surgical resection, with systemic treatment is essential to improve the prognosis.

CGP has emerged as a critical tool for identifying individual tumor vulnerabilities and guiding the selection of systemic therapies.^4)^ Herein, we report the case of a patient with a solitary brain metastasis from ICC who achieved a favorable outcome using a multimodal strategy. The therapeutic plan was critically informed by the CGP, which revealed a high tumor mutational burden (TMB-High), providing a clear rationale for the use of immunotherapy.

## CASE PRESENTATION

**Fig. 1:**
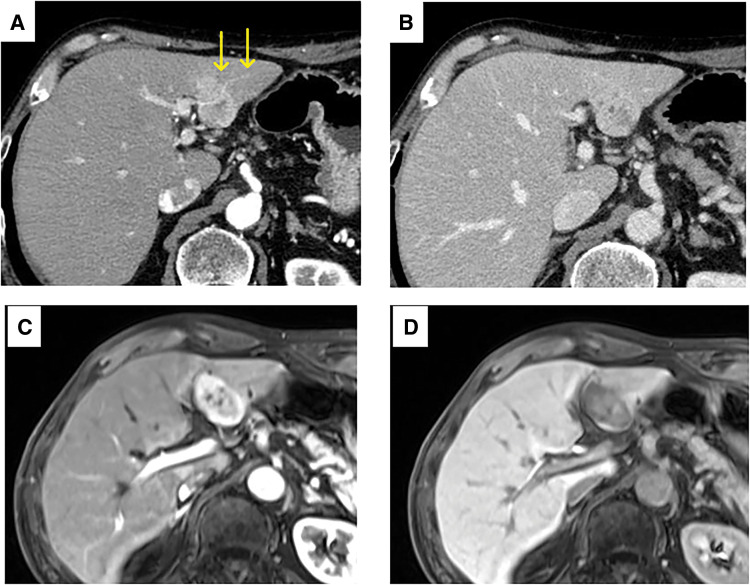
Preoperative imaging findings of the ICC. (**A**) Contrast-enhanced CT in the arterial phase reveals a tumor with peripheral ring enhancement and a penetrating vessel sign (arrows). (**B**) Delayed-phase CT demonstrates that the tumor shows iso-enhancement compared with the adjacent parenchyma. (**C**) Arterial phase of contrast-enhanced MRI also shows peripheral hyperenhancement. (**D**) Hepatobiliary phase MRI reveals a clear signal defect in the tumor. ICC, intrahepatic cholangiocarcinoma

**Fig. 2:**
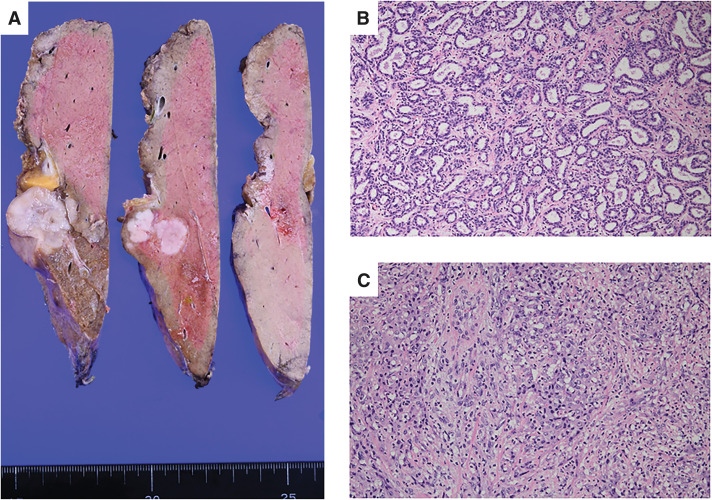
Macroscopic and microscopic findings of the resected hepatic tumor. (**A**) Macroscopic appearance of the resected specimen, showing a whitish, lobulated tumor mass. (**B**) Well-differentiated component, showing an organized pattern of tubular and glandular structures (hematoxylin and eosin stain, ×20). (**C**) Poorly differentiated component, showing a disorganized, solid sheet-like growth pattern with a loss of glandular architecture (hematoxylin and eosin stain, ×20).

A 64-year-old man with a history of lung cancer, pT2aN0M0, pStage IB according to the UICC 8th edition, for which he had undergone resection 4 years prior, presented for routine follow-up. Although the patient was asymptomatic, a hepatic mass was incidentally discovered on CT. Contrast-enhanced CT revealed a 36-mm mass characterized by peripheral ring enhancement in the arterial phase and a penetrating vessel sign (**Fig. 1A**). In the delayed phase, it showed iso-enhancement relative to the surrounding liver parenchyma (**Fig. 1B**). The tumor invaded the root of the Glissonian pedicle for segment 3 and was accompanied by a daughter nodule and enlarged hilar lymph nodes. On contrast-enhanced MRI, the lesion demonstrated peripheral hyperenhancement in the arterial phase (**Fig. 1C**) and washout in the portal venous phase. It showed a distinct signal defect in the hepatobiliary phase (**Fig. 1D**). He was diagnosed with ICC and underwent a laparoscopic left hepatectomy and hilar lymph node sampling. The operative duration was 358 minutes, and the blood loss was 102 mL. No blood transfusion was required. His postoperative course was uneventful, and he was discharged without complications. Macroscopically, the tumor was a lobulated mass measuring 38 × 30 mm (**Fig. 2A**). Microscopically, the tumor exhibited a mixed histology, composed of both well-differentiated (**Fig. 2B**) and poorly differentiated (**Fig. 2C**) components. The well-differentiated component was characterized by clearly formed tubular structures lined by cuboidal cells with minimal atypia. In contrast, the poorly differentiated component displayed a solid growth pattern with a loss of glandular structures and marked cellular pleomorphism. Immunohistochemically, the tumor cells were positive for CK7, partially positive for CA19-9, and negative for both alpha-fetopeotein and CD10. The final diagnosis was ICC, classified as pT2N0M0 pStage II according to the UICC 8th edition. He began adjuvant chemotherapy with oral tegafur/gimeracil/oteracil potassium (100 mg/day); however, the treatment was discontinued after the 1st course due to severe fatigue.

**Fig. 3:**
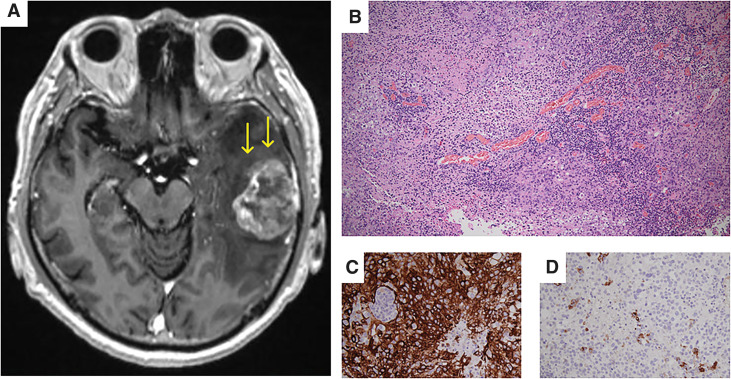
MRI and microscopic findings of the resected brain tumor. (**A**) Contrast-enhanced brain MRI reveals a solitary, ring-enhancing mass measuring 39 × 29 mm in the left temporal lobe. (**B**) Histological examination of the resected brain tumor reveals a poorly differentiated adenocarcinoma, which was morphologically similar to the previously resected primary ICC (×10). (**C**) CK7 staining is positive (×20). (**D**) CA19-9 staining is focally positive (×20). CA19-9, carbohydrate antigen 19-9; ICC, intrahepatic cholangiocarcinoma

At 15 months after hepatectomy, he reported difficulty with recent memory. Contrast-enhanced brain MRI revealed a solitary, ring-enhancing mass measuring 39 × 29 mm in the left temporal lobe (**Fig. 3A**). Fluorodeoxyglucose-PET did not reveal any abnormal uptake suggestive of distant metastasis or recurrence, other than the intracranial lesion. He underwent surgical resection of the tumor on suspicion of a brain metastasis from either lung cancer or ICC. Histological examination of the resected brain tumor revealed a poorly differentiated adenocarcinoma, which was morphologically similar to the previously resected primary ICC (**Fig. 3B**). On immunohistochemical staining, the brain metastasis was positive for CK7 (**Fig. 3C**), negative for carcinoembryonic antigen, and focally positive for CA19-9 (**Fig. 3D**). This profile was consistent with that of the primary hepatic lesion. Therefore, the brain lesion was diagnosed as a metastasis from the ICC. PD-L1 testing was not performed because PD-L1 staining is not yet routinely available at our institution and would have required outsourcing the specimen to an external laboratory.

After craniotomy, the patient’s neurological symptoms promptly improved. He began systemic therapy and received 8 cycles of gemcitabine (1000 mg/m^2^ on days 1 and 8) and cisplatin (25 mg/m^2^ on days 1 and 8), and durvalumab (1500 mg) every 3 weeks. In parallel, FoundationOne CDx (Foundation Medicine, Boston, MA, USA) was performed on the resected brain specimen. The analysis did not detect actionable alterations, such as *FGFR2* fusions or *IDH1/2* mutations. Mutations were identified in several other genes, including *CRKL*, *MAPK1*, *PTPRO*, *STK11*, and *TP53*; however, none of these genes were considered directly targetable (**Table 1**). The results showed a TMB-High (23 mutations/Mb) and microsatellite stability. After completing the combination therapy, the patient continued maintenance durvalumab monotherapy. The patient remained disease-free with no signs of recurrence on brain MRI and chest-abdominal CT at 8 months post-surgery.

**Table 1:** Result of comprehensive genomic profiling

Altered gene	Variant
*CRKL*	Amplification
*MAPK1*	Amplification
*PTPRO*	Splice site 2305-1G>A
*STK11*	Loss
*TP53*	H193R

## DISCUSSION

We report a patient with a solitary ICC brain metastasis who achieved a favorable outcome. Management involved a sequential, multimodal approach, beginning with resection of the brain tumor for both diagnostic and therapeutic purposes. Following pathological confirmation of ICC metastasis, systemic therapy was initiated. In parallel, CGP was performed, and the results revealed TMB-High, providing a strong molecular rationale that supported the validity of the chosen regimen containing an ICI. This case illustrates how integrating timely local control with biomarker-informed systemic therapy can restore expectations in a historically grim clinical scenario. The patient achieved disease-free survival at 8 months postoperatively, making this case a valuable example of how a modern, adaptive treatment strategy can potentially overcome the historically poor prognosis of this condition. This demonstrates the practical implementation of precision oncology in real-world BTC care.

Brain metastasis of BTC is a rare clinical condition. A large cohort study has reported that brain metastases are found in 1.4% of patients with BTC.^3)^ It usually occurs in the terminal stage of the tumor, with an overall survival of 1.8–3.7 months.^3,5)^ However, there are a few reported cases, such as ours, where solitary brain metastasis without other organ involvement occurred after surgery for BTC. To contextualize our case within the existing literature, we summarized previously reported cases of solitary brain metastases from biliary tract cancer (**Table 2**).^6–8)^ Only 3 cases have been documented worldwide and all reported patients experienced relatively favorable survival after the diagnosis of brain metastasis. A study of brain metastases from various solid tumors has identified age, the number of brain lesions, neurologic performance status, and the presence of extracranial metastases as prognostic factors.^9)^ Although this cohort did not include patients with BTC, such study has suggested that solitary brain metastasis itself may be associated with a relatively favorable prognosis. Genomic data on these rare tumors are scarce. To our knowledge, including our own report, CGP has only been performed on 2 cases. Notably, our case is the first to demonstrate a TMB-High phenotype in ICC brain metastasis, directly influencing the choice of immunotherapy.

**Table 2:** Literature review of solitary brain metastasis after biliary tract cancer resection

Reported year	Age*/Sex	Diagnosis	Presenting symptom	BM resection	BM radiation	Time between primary tumor and BM (month)	CGP	Systemic therapy	OS**
1991	68/F	GBC	Convulsion	Yes	No	5	No	Cisplatin	48 (Alive)
2011	60/M	ICC	–	Yes	SRS	10	No	Tegafur and uracil	74 (Alive)
2022	54/F	ICC	Limb weakness	No	No	3	KIT, TP53, PDGFR	Camrelizumab and lenvatinib	17.5 (Alive )
Our case	64/M	ICC	Difficulty with recent memory	Yes	No	15	TP53, TMB-High	Gemcitabine, cisplatin, and durvalumab	8 (Alive)

* Age at primary diagnosis, years.

**Time from BM to last follow-up (month).

BM, brain metastasis; CGP, comprehensive genomic profiling; F, female; GBC, gallbladder carcinoma; ICC, intrahepatic cholangiocarcinoma; M, male; OS, overall survival; SRS, stereotactic radiosurgery

Surgical resection is the preferred initial strategy for solitary brain lesions primarily because it offers curative potential. This is particularly relevant in cases of oligometastasis, a condition in which targeted loco-regional therapy improves survival.^10)^ While the criteria for oligometastasis in BTC are still evolving, a study by Morino et al. suggested that patients with single-organ recurrence, a maximum of 3 tumors, and a latency of >1 year to recurrence may benefit from such an approach.^11)^ Therefore, the presentation of a solitary brain metastasis 15 months post-treatment in our case was consistent with oligometastasis, making the surgical intervention a retrospectively appropriate choice. Second, it provides therapeutic relief by decompressing large symptomatic masses. Although radiation therapy can also be used for palliation, its effect on relieving the mass effect is typically delayed compared with surgery.^12)^ Third, it provides diagnostic certainty by pathologically differentiating the tumor from a patient’s prior lung cancer. Given the possibility of metastatic lung adenocarcinoma in this patient, tissue confirmation was indispensable; importantly, it also enabled CGP on the metastatic focus, which would not have been possible with empirical radiation alone.

Systemic therapy is indicated after a successful resection to address the risk of micrometastatic recurrence. In the present case, gemcitabine, cisplatin, and durvalumab were administered after resection of the solitary brain metastasis. In other malignancies such as melanoma and renal cell carcinoma, adjuvant ICI monotherapy after curative-intent surgery, including resection of stage III/IV lesions, has been shown to reduce the risk of recurrence.^13,14)^ However, no studies have demonstrated the efficacy of a combination of chemotherapy and immune checkpoint inhibition as adjuvant treatment after complete resection of metastatic disease. Given that stage IV BTC generally carries a poor prognosis and that occult micrometastatic disease was highly suspected, we considered postoperative systemic therapy to be necessary and therefore adopted gemcitabine, cisplatin, and durvalumab in accordance with the TOPAZ-1 regimen,^15)^ integrating evidence from this trial with patient-specific molecular findings. From a biological standpoint, TMB-High increases the probability of neoantigen generation, thereby augmenting tumor immunogenicity and enhancing sensitivity to PD-L1 blockade. Concurrent CGP revealed TMB-High, which provided a direct molecular rationale for the inclusion of the ICI durvalumab. TMB-High remains uncommon in BTC and is typically reported at low single-digit frequencies in published cohorts.^16)^ Therefore, its identification represents a meaningful therapeutic opportunity for a disease that lacks broadly effective targeted options. While the precise contribution of this regimen to the patient’s disease-free survival cannot be quantified, the identification of a TMB-High status also holds future strategic value, confirming that options such as pembrolizumab may be available upon recurrence. Although the metastatic lesion was microsatellite stable, its coexistence with TMB-High indicates that hypermutation-driven immunogenicity may occur independently of mismatch repair deficiency in selected BTCs.

However, the likelihood of response to ICIs is not determined by TMB alone. Increasing evidence suggests that PD-L1 expression and the broader tumor immune microenvironment—including the density and spatial distribution of tumor-infiltrating lymphocytes—also modulate the efficacy of checkpoint blockade.^17)^ In the present case, we did not evaluate PD-L1 expression, tumor-infiltrating lymphocyte density, or their spatial organization, and therefore the immune context in which durvalumab was administered cannot be fully characterized. Nevertheless, we consider that incorporating such tumor immune profiling in addition to TMB assessment may become an important element of future strategies for predicting and interpreting responses to ICIs.

CGP drives personalized management in modern BTC. Its clinical utility is based on the identification of 2 main categories of therapeutic targets. The first category includes specific “actionable” driver mutations, such as *FGFR2* fusions^18)^ or *IDH1* mutations,^19)^ that make tumors sensitive to targeted therapies. The present case belongs to the 2nd category, which involves the discovery of other biomarkers to guide treatment selection. Here, although no classic “actionable” driver was identified, the determination of a TMB-High status was highly informative for guiding the use of immunotherapy. Crucially, the clinically actionable information in this case was derived from the metastatic tissue, highlighting spatial and temporal tumor heterogeneity and supporting re-biopsy at progression or relapse rather than relying solely on archival primary specimens. Additionally, several co-mutations (e.g., *TP53* and *STK11*) have been identified, but their predictive value for ICI response in BTC remains uncertain. Their documentation adds to an emerging mutational context that future studies may use to refine biomarker models beyond TMB alone.

Our experience suggests a pragmatic framework for selected patients with oligometastatic ICC presenting with a solitary brain lesion: (i) pursue surgical resection when feasible to achieve immediate mass control and obtain tissue, (ii) apply CGP to the metastatic specimen to uncover biomarkers, such as TMB-High or microsatellite instability-high, and (iii) integrate biomarker-guided systemic therapy (gemcitabine/cisplatin with PD-L1 blockade per TOPAZ-1)^15)^ while considering maintenance ICI in responders.

This integrated approach may yield outcomes superior to the historical expectations for BTC brain metastasis and merits prospective evaluation; however, it has some limitations. First, the multimodal nature of treatment makes it impossible to ascertain the independent contribution of each component (surgery, chemotherapy, or immunotherapy) to the outcome. Second, genomic profiling was limited to metastatic lesions; without data from the primary tumor, an analysis of clonal evolution, such as changes in TMB status during metastasis, could not be performed. Third, while the disease-free survival period is encouraging, a longer follow-up is required to assess the long-term durability of the response. Furthermore, we did not incorporate longitudinal circulating tumor DNA monitoring, which may have provided complementary insights into minimal residual disease and molecular response dynamics.

## CONCLUSIONS

Our findings support a treatment paradigm in which aggressive local therapy for carefully selected oligometastatic ICC is coupled with CGP-guided immunotherapy. A systematic collection of such cases, ideally within registries or prospective studies, will be essential to validate TMB-High as a predictive biomarker for BTC and to define the patient subsets most likely to benefit from this precision-oncology strategy.

## DECLARATIONS

### Availability of data and materials

The authors declare that all the data related to this study are available in the manuscript.
